# Burden of Parkinsonism and Parkinson’s Disease on Health Service Use and Outcomes in Latin America

**DOI:** 10.3233/JPD-230114

**Published:** 2023-11-03

**Authors:** Dani J. Kim, Ana M. Rodriguez-Salgado, Juan J. Llibre-Rodriguez, Isaac Acosta, Ana Luisa Sosa, Daisy Acosta, Ivonne Z. Jimenez-Velasquez, Mariella Guerra, Aquiles Salas, Christine Jeyachandran, Ricardo López-Contreras, Heike Hesse, Caroline Tanner, Jorge J. Llibre-Guerra, Matthew Prina

**Affiliations:** a Health Service and Population Research Department, Institute of Psychiatry, Psychology, and Neuroscience, King’s College London, London, UK; b Population Health Sciences Institute, Faculty of Medical Sciences, Newcastle University, Newcastle upon Tyne, UK; c Global Brain Health Institute, University of San Francisco, San Francisco, CA, USA; d Dementia Research Unit, Medical University of Havana, Havana, Cuba; e Laboratory of the Dementias, National Institute of Neurology and Neurosurgery, Mexico City, Mexico; f National Autonomous University of Mexico, Mexico City, Mexico; g Universidad Nacional Pedro Henriquez Ureña (UNPHU), Internal Medicine Department, Geriatric Section, Santo Domingo, Dominican Republic; h Internal Medicine Department, Geriatrics Program, School of Medicine, Medical Sciences Campus, University of Puerto Rico, San Juan, Puerto Rico; i Instituto de la Memoria Depresion y Enfermedades de Riesgo IMEDER, Lima, Perú; j Medicine Department, Caracas University Hospital, Faculty of Medicine, Universidad Central de Venezuela, Caracas, Venezuela; k Faculty of Medicine and Health, University of New South Wales, Sydney, NSW, Australia; l Memory Clinic, Neurology Service, Salvadoran Social Security Institute, San Salvador, El Salvador; m Observatorio Covid-19, Universidad Tecnológica Centroamericana, Tegucigalpa, Honduras; n Department of Neurology, Weill Institute for Neurosciences, University of California-San Francisco, San Francisco, CA, USA; o Department of Neurology, Washington University School of Medicine in St. Louis, St. Louis, MO, USA

**Keywords:** Parkinsonian disorders, Latin America, health services administration, patient care, mortality

## Abstract

**Background:**

Little is known about the burden of parkinsonism and Parkinson’s disease (PD) in Latin America. Better understanding of health service use and clinical outcomes in PD is needed to improve its prognosis.

**Objective:**

The aim of the study was to estimate the burden of parkinsonism and PD in six Latin American countries.

**Methods:**

12,865 participants aged 65 years and older from the 10/66 population-based cohort study were analysed. Baseline assessments were conducted in 2003–2007 and followed-up 4 years later. Parkinsonism and PD were defined using current clinical criteria or self-reported diagnosis. Logistic regression models assessed the association between parkinsonism/PD with baseline health service use (community-based care or hospitalisation in the last 3 months) and Cox proportional hazards regression models with incident dependency (subjective assessment by interviewer based on informant interview) and mortality. Separate analyses for each country were combined via fixed effect meta-analysis.

**Results:**

At baseline, the prevalence of parkinsonism and PD was 7.9% (*n* = 934) and 2.6% (*n* = 317), respectively. Only parkinsonism was associated with hospital admission at baseline (OR 1.89, 95% CI 1.30–2.74). Among 7,296 participants without dependency at baseline, parkinsonism (HR 2.34, 95% CI 1.81–3.03) and PD (2.10, 1.37–3.24) were associated with incident dependency. Among 10,315 participants with vital status, parkinsonism (1.73, 1.50–1.99) and PD (1.38, 1.07–1.78) were associated with mortality. The Higgins I^2^ tests showed low to moderate levels of heterogeneity across countries.

**Conclusions:**

Our findings show that older people with parkinsonism or PD living in Latin America have higher risks of developing dependency and mortality but may have limited access to health services.

## INTRODUCTION

Parkinson’s disease (PD) is a neurological disorder that affects around 8.5 million people worldwide [1] and the fastest growing neurological condition after dementia [2]. The prevalence of PD has more than doubled in 26 years from 2.5 million in 1990 to 6.1 million in 2016 [3]. PD results in a slowly accumulating disability, long-term dependency on a carer [4], and considerable health and socioeconomic burden in patients. For instance, PD is associated with higher rates of hospital admission and health service use [5–7], and nearly 3-fold greater risk of dying within the first 10 years of diagnosis (hazard ratios 2.48, 95% CI 1.55–3.95, *p* < 0.001) compared to the general population [8]. Also, it was estimated that PD contributed to nearly 6 million disability-adjusted life years in 2019 [9]. Thus, the rising prevalence and the burden associated with PD suggests the anticipated higher need for health care services and morbidity due to PD is greater than any other neurological disorder.

Much of the parkinsonism and PD research to date have focused on high-income countries and research in low-and-middle income countries (LMICs), including the Latin America region, is lacking. Recent literature suggests likely differences in the epidemiology of PD by region. For instance, PD, unlike other non-communicable diseases, is more prevalent in countries with a high socio-demographic index (SDI) and rapidly increasing in middle SDI countries, which is believed to be due to higher exposure to environmental PD risk factors related to industrialization [3]. Thus, countries that are at different stages of development, with varying levels of public awareness in PD and accessibility to PD treatment, may have a different PD prevalence and burden compared to Western countries. To our knowledge, the burden of parkinsonism or PD has not been estimated in Latin America countries.

The aim of the present study was to estimate the burden of parkinsonism and PD in 6 Latin American countries: Cuba, Dominican Republic, Peru, Venezuela, Mexico, and Puerto Rico. A large cross-country prospective cohort study from the 10/66 Dementia Research Group [10], which collected data at baseline and follow-up surveys, was used to investigate the association between parkinsonism and PD with the following outcome measures: health service use (community health service and hospital admission), incident dependency, and all-cause mortality.

## METHODS

The study was reported according to the STROBE checklist for cohort studies (Supplementary Table 1) [11].

### Setting and participants

Data from the present study originate from the 10/66 Dementia Research Group population-based cohort study [10]. The 10/66 cohort comprises adults aged 65 years and over living in 10 LMICs (India, China, Nigeria, Cuba, Dominican Republic, Brazil, Venezuela, Mexico, Peru, and Argentina) [12]. These sites were selected purposively to maximize their accessibility and relationship with local research groups and stakeholders [10]. While 4 countries (China, India, Peru, and Mexico) included separate urban and rural catchment sites, the remaining 6 countries included data from urban areas only [12]. The rural sites were remote areas with low population density and an agricultural lifestyle, while urban sites were areas with low or mixed socioeconomic status households (areas that were predominantly middle-class or high-income earners were excluded) [12]. The sample size calculations for each country have been reported in the 10/66 study protocol [12]. Eligible participants were identified by door-knocking all households in the catchment area [10].

The baseline data was collected between 2003 and 2007 (in Puerto Rico, data was collected between 2006 and 2008) [10]. Participants were followed-up after approximately 4 years (from 2007 to 2011) for a repeat assessment of the baseline survey [10, 12]. The response rates for the baseline surveys ranged from 74% to 98% [12]. All participants underwent a full interview including physical and biological assessments, lasting around 2-3 hours in their own homes [12]. A standardized operating procedures manual was used to train each of the 4–10 interviewers of the study centers, who were generally lay graduates (or medical doctors in Cuba), on the study protocol, standard structured interviewing techniques, and the cognitive and neurological examination [12]. All study participants gave written informed consent. Local ethics committees and the King’s College London Research Ethics Committee approved the study.

For the present study, 6 Latin America countries were included: Cuba (*n* = 2,944), Dominican Republic (*n* = 2,011), Peru (*n* = 1,933), Venezuela (*n* = 1,965), Mexico (*n* = 2,003), and Puerto Rico (*n* = 2,009). Analyses were carried out using 3 different analytical cohorts. First, the cross-sectional analyses included the 12,865 baseline participants; second, the dependency cohort included 7,296 participants who were not considered dependent at baseline, were re-interviewed at follow-up, and had complete data to assess dependency; and lastly, the mortality cohort included 10,315 participants whose vital status was ascertained at follow-up. The baseline characteristics according to vital status ascertainment were compared.

### Measures

The 10/66 cohort study was initially developed to investigate the prevalence, incidence, and impact of dementia across LMICs [12]. Accordingly, the survey involved a comprehensive assessment of a wide range of health-related aspects including information of demographics, chronic diseases, disability, health service utilization, and socioeconomic status. The 10/66 study also included physical and neurological examinations and an assessment of neurological diseases. The interviews and tests were undertaken by trained research staff using standardized study protocols and procedures across study sites. Full details of these protocols and procedures are available elsewhere [10, 12]. Here, we describe the relevant variables for this paper.

### Diagnosis of parkinsonism and Parkinson’s disease

All participants underwent a comprehensive interview, including a structure interview, a physical and neurological examination, and an informant interview [12]. The interviewers selected key informants, usually co-residents, family members, and caregivers, who they considered to be the most knowledgeable about the current circumstances of the older person [10]. This comprehensive interview obtained data on self-reported chronic diseases (e.g., stroke) and neurological symptoms (e.g., tremor), which permitted the diagnosis of parkinsonism and PD using an algorithm based on current clinical criteria [13].

Parkinsonism and PD was defined according to the United Kingdom Parkinson’s Disease Society Brain Research Centre criteria (Supplementary Table 2) [14]. First, parkinsonism was diagnosed as the presence of bradykinesia (slowness of voluntary movement with progressive difficulty performing repetitive actions) and at least one of the following: rest tremor, muscular rigidity, or postural instability not caused by primary visual, vestibular, cerebellar, or proprioceptive dysfunction. Subsequently, PD was diagnosed when there was at least 3 supportive criteria (e.g., rest tremor, progressive disorder, and asymmetry) that favor a PD diagnosis and no red flags (e.g., repeated strokes, supranuclear gaze palsy, cerebellar signs, cerebral tumor, and severe autonomic involvement) that argue against a PD diagnosis [14, 15]. This diagnostic algorithm has been recommended for use in epidemiological studies [16]. Additionally, the sensitivity (94% for parkinsonism and 86% for PD) and specificity (97% for parkinsonism and 99% for PD) of the diagnostic algorithm was estimated in the Cuba sample using clinical diagnoses by two neurologists as the reference standard [13]. Lastly, PD diagnosis was also supplemented by self-reported diagnoses of PD obtained from the structured interviews.

### Definition of confounders

The following covariates were included in the analysis: age (years), sex (male or female), educational level (none, did not complete primary, completed primary, secondary, or tertiary education), and the number of physical illnesses (none, one to two, three or more). The number of physical illnesses was defined from a list of nine illnesses: arthritis or rheumatism; persistent cough; breathlessness, difficulty breathing or asthma; high blood pressure; heart trouble or angina; stomach or intestine problems; faints or blackouts; paralysis, weakness, or loss of one leg or arm; skin disorders such as pressure sores, leg ulcers or severe burns.

### Definition of outcomes

Health service use was measured using the LMIC-adapted Client Services Receipt Inventory at baseline [17]. Participants or a key informant were asked to recall whether they 1) had any contact with community health services (any one of: primary care, hospital doctor, private doctor, dentist, traditional healer, or other services) or 2) had been admitted to hospital in the previous three months [18].

Dependency was determined using a series of open-ended questions to an informant at baseline and follow-up [19, 20]. The interviewer then coded whether the participant required no care, care some of the time, or care much of the time based on their perception of need for care [10, 21]. Incident dependency was defined as needing care some of the time or much of the time at follow-up among the dependency cohort.

A mortality sweep was conducted on the whole baseline cohort at follow-up [19, 20]. A verbal autopsy was carried out in appropriate informants of deceased participants to ascertain the date of death [10].

### Statistical analyses

The present study used the 10/66 baseline and incidence data. The baseline characteristics of participants were reported for individual countries and overall. Logistic regression models were used to assess the cross-sectional associations between parkinsonism or PD and health service use (community health service or hospital admission) at baseline. Cox proportional hazards models were used to assess the prospective associations between parkinsonism or PD and incident dependency (using the dependency cohort) and mortality (using the mortality cohort). The proportional hazards assumption for the Cox regression models was tested and violation of the assumption was accounted for by stratifying models. Participants were censored at the date of event (death or incident dependency) or the last date of follow-up. The precise date of dependency was not captured in the 10/66 survey design; therefore, the date of event was estimated as the median length of period between the baseline and follow-up interview for participants with incident dependency. For all analyses, both crude models and models adjusted for age, sex, education level, and the number of physical illnesses were fitted. Models were fitted separately for each country and combined via a fixed effect meta-analysis, estimating the magnitude of heterogeneity using Higgins I^2^ statistic. As a sensitivity analysis, we repeated the analyses using the PD definition based on the diagnostic algorithm only (i.e., excluding self-reported PD diagnoses). Complete case analyses were conducted. All analyses were performed in R version 4.2.1.

## RESULTS

### Cohort characteristics

The baseline characteristics of the study cohort are presented in Table 1. Among 12,865 participants aged ≥65 years, 35.5% were male and the mean age (standard deviation, SD) was 74.7 (7.24) years. Nearly half were married or cohabiting (45.7%), had attended some years or completed primary education (58%), and had one or two illnesses (42.7%). The prevalence of parkinsonism and PD was 7.9% (*n* = 934) and 2.6% (*n* = 317), respectively. The breakdown of the PD diagnosis by source (UK Parkinson’s Disease Society Brain Bank diagnostic criteria or self-reported diagnosis) is shown in Supplementary Table 3. Of the 317 cases of PD identified, 100 (31.5%) had a previous self-reported diagnosis of PD. Community health service use in the 3 months leading up to the study interview was reported by more than half (56.8%) of the participants and hospital admission by 3%. Dependency was present in 10.5% (1,352) of participants at baseline. Of the 10,315 participants whose vital status was ascertained, 1,730 deaths (16.8%) were recorded after an average (SD) follow-up of 3.8 (1.24) years.

**Table 1:** Cohort characteristics at baseline, overall and by individual country

N (%) or Mean (SD)	Overall	Cuba	Dominican Republic	Peru	Venezuela	Mexico	Puerto Rico
Total	12,865	2,944	2,011	1,933	1,965	2,003	2,009
Age	74.74 (7.24)	75.08 (7.04)	75.25 (7.51)	74.80 (7.36)	72.49 (6.91)	74.30 (6.66)	76.35 (7.42)
Male sex	4,568 (35.5)	1,031 (35.0)	684 (34.0)	750 (38.8)	713 (36.3)	735 (36.7)	655 (32.7)
Marital status
Never married	1,044 (8.2)	275 (9.4)	139 (7.0)	213 (11.1)	189 (9.8)	105 (5.2)	123 (6.1)
Married/cohabiting	5,845 (45.7)	1,271 (43.3)	586 (29.4)	1,092 (56.8)	921 (48.0)	1,008 (50.3)	967 (48.3)
Widowed	4,245 (33.2)	928 (31.6)	806 (40.4)	524 (27.3)	549 (28.6)	766 (38.3)	672 (33.6)
Divorced/ separated	1,644 (12.9)	462 (15.7)	465 (23.3)	93 (4.8)	261 (13.6)	123 (6.1)	240 (12.0)
Education level
None	1,370 (10.7)	75 (2.6)	392 (19.7)	121 (6.3)	156 (8.1)	554 (27.7)	72 (3.6)
Some, did not complete primary	3,606 (28.2)	655 (22.3)	1,022 (51.3)	231 (12.1)	445 (23.1)	864 (43.2)	389 (19.4)
Completed primary	3,807 (29.8)	979 (33.3)	370 (18.6)	727 (37.9)	965 (50.1)	351 (17.5)	415 (20.7)
Completed secondary	2,483 (19.4)	728 (24.8)	135 (6.8)	517 (27.0)	266 (13.8)	124 (6.2)	713 (35.5)
Tertiary (college)	1,504 (11.8)	499 (17.0)	73 (3.7)	321 (16.7)	93 (4.8)	108 (5.4)	410 (20.4)
Number of assets
1st quartile – least assets	2,226 (17.3)	451 (15.4)	643 (32.1)	155 (8.0)	48 (2.4)	376 (18.8)	553 (27.5)
2nd quartile	4,596 (35.8)	876 (29.8)	444 (22.1)	1,134 (58.7)	1,298 (66.1)	844 (42.1)	0 (0.0)
3rd quartile	3,608 (28.1)	1,073 (36.5)	733 (36.5)	181 (9.4)	0 (0.0)	165 (8.2)	1,456 (72.5)
4th quartile – most assets	2,422 (18.8)	536 (18.3)	186 (9.3)	463 (24.0)	619 (31.5)	618 (30.9)	0 (0.0)
Number of illnesses
No illnesses	5,066 (39.5)	1,289 (43.9)	599 (29.8)	887 (45.9)	748 (38.7)	835 (41.7)	708 (35.4)
One to two illnesses	5,467 (42.7)	1,357 (46.2)	945 (47.0)	780 (40.4)	695 (36.0)	825 (41.2)	865 (43.2)
Three or more illnesses	2,282 (17.8)	292 (9.9)	465 (23.1)	264 (13.7)	489 (25.3)	343 (17.1)	429 (21.4)
Parkinsonism	934 (7.9)	184 (6.3)	201 (10.5)	144 (7.5)	89 (6.3)	178 (8.9)	138 (8.6)
Parkinson’s disease*	317 (2.6)	97 (3.3)	48 (2.5)	39 (2.0)	45 (3.0)	50 (2.5)	38 (2.2)
Health service use
One or more community health service use	7,305 (56.8)	1,420 (48.2)	944 (46.9)	811 (42.0)	1,188 (60.5)	1,303 (65.1)	1,639 (81.6)
Hospital admission	379 (3.0)	62 (2.1)	61 (3.0)	34 (1.8)	77 (4.0)	38 (1.9)	107 (5.3)
Dependency (need for care)
Much of the time	763 (6.1)	169 (6.5)	143 (7.1)	85 (4.4)	98 (5.0)	86 (4.3)	182 (9.1)
Some of the time	589 (4.7)	92 (3.5)	94 (4.7)	76 (3.9)	111 (5.7)	110 (5.5)	106 (5.3)
Does not need care	11,150 (89.2)	2,335 (89.9)	1,770 (88.2)	1,770 (91.7)	1,754 (89.4)	1,807 (90.2)	1,714 (85.6)
Vital status ascertained	10,315 (87.3)	1,749 (92.3)	1,706 (84.8)	1,752 (90.6)	1,697 (86.4)	1,844 (92.1)	1,567 (78.0)
Deaths among this group	1,730 (16.8)	404 (23.1)	467 (27.4)	152 (8.7)	200 (11.8)	209 (11.3)	298 (19.0)
Follow-up (y)	3.80 (1.24)	4.31 (1.36)	4.37 (1.43)	3.06 (0.79)	4.14 (0.96)	2.91 (0.54)	4.13 (1.17)

^*^Parkinson’s disease was defined using either self-reported diagnosis or the United Kingdom Parkinson’s Disease Society Brain Bank (UKPDSBB) criteria.

The number of participants included in each country ranged from 1,933 (Peru) to 2,944 (Cuba). The mean age and gender distribution were similar across studies, but some variation in demographic factors was observed. For instance, Dominican Republic had one of the highest levels of participants who had divorced/separated (23.3%), (in)complete primary education (66.9%), one or two illnesses (47.0%), parkinsonism (10.5%), dependency (11.8%), and all-cause mortality (27.4%). Puerto Rico had the highest levels of community health service use (81.6%), hospital admission (5.3%), dependency (14.4%), and relatively high levels of mortality (19.0%). Conversely, Peru had one of the lowest levels of participants with community health service use (42.0%), hospital admission (1.8%), dependency (8.3%), PD (2.0%), and mortality (8.7%).

The flow diagram of the study participants for the present study is shown in Supplementary Figure 1. The baseline characteristics of the dependency (*n* = 7,296) and mortality cohort (*n* = 10,315) are presented in Supplementary Table 4 and Supplementary Table 5, respectively. The difference in baseline characteristics between participants with and without their vital status ascertained are shown in Supplementary Table 6. In the dependency cohort, 536 participants (7.3%) became dependent at follow-up.

### Association of parkinsonism and PD with outcomes

The crude associations between parkinsonism and PD with outcomes are reported in Supplementary Table 7. Participants at baseline with and without parkinsonism had similar level of community health service use (58.4% vs. 56.0%; *p* = 0.168), but the frequency of hospital admission (4.3% vs. 2.5%; *p* = 0.001), incident dependency (20.5% vs. 6.3%; *p* < 0.001), and mortality (33.8% vs. 14.3%; *p* < 0.001) was higher in those with parkinsonism. PD cases and non-cases had similar level of hospital admission (2.5% vs. 2.8%; *p* = 0.931), but the frequency of community health service use (62.5% vs. 56.1%; *p* = 0.029), incident dependency (18.5% vs. 7.1%; *p* < 0.001), and mortality (27.4% vs. 16.3%; *p* < 0.001) was higher in PD cases.

**Fig. 1:**
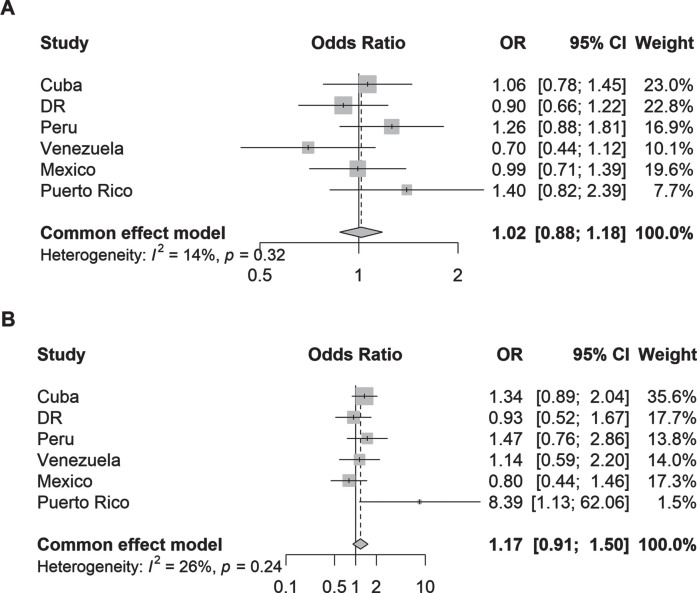
Odds ratios (95% CI) of the association between (A) parkinsonism and (B) Parkinson’s disease and one or more community health services used by country. Logistic regression models were adjusted for age, sex, education, and number of illnesses (none; 1-2 illnesses; 3 or more illnesses).

In the logistic regression models, neither parkinsonism (adjusted odds ratio [aOR] 1.02, 95% CI 0.88–1.18; I^2^ = 14%) nor PD (aOR 1.17, 95% CI 0.91–1.50; I^2^ = 26) was associated with community health service use after adjustment for age, sex, education level, and the number of illnesses (Fig. 1). Individual country analyses also showed no associations except for Puerto Rico for PD. Given the lack of association between parkinsonism/PD and community health service use, a *post-hoc* sensitivity analysis was carried out to determine whether the individual community health service components were associated with parkinsonism or PD. Country-specific analyses were not carried out due to insufficient power. The adjusted ORs between individual health service components (Supplementary Table 8) showed that none of the individual community health service was associated with parkinsonism or PD apart from PD and primary care (aOR 1.31, 95% CI 1.01–1.69).

**Fig. 2:**
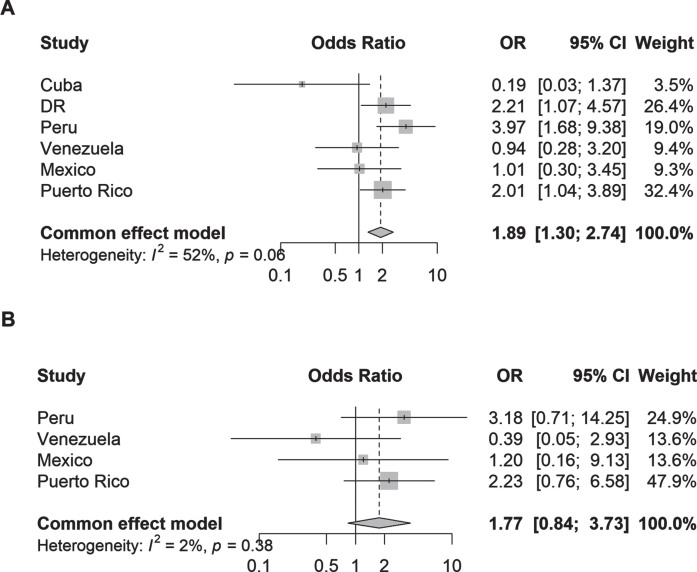
Odds ratios (95% CI) of the association between (A) parkinsonism and (B) Parkinson’s disease and hospital admission by country. Logistic regression models were adjusted for age, sex, education, and number of illnesses (none; 1-2 illnesses; 3 or more illnesses); Plot B excludes data from Cuba and Dominican Republic due to zero events, thus data from these countries did not contribute to the fixed-effects model.

Parkinsonism (aOR 1.89, 95% CI 1.30–2.74; I^2^ = 52%) but not PD (aOR 1.77, 95% CI 0.84–3.73, I^2^ = 2%) was associated with hospital admission after adjustment for covariates compared to non-cases (Fig. 2). The analysis of individual countries showed that significant associations between parkinsonism and hospital admission was present in Dominican Republic (aOR 2.21, 95% CI 1.07–4.57), Peru (aOR 3.97, 95% CI 1.68–9.38), and Puerto Rico (aOR 2.01, 95% CI 1.04–3.89); and none were found for PD and hospital admission.

**Fig. 3:**
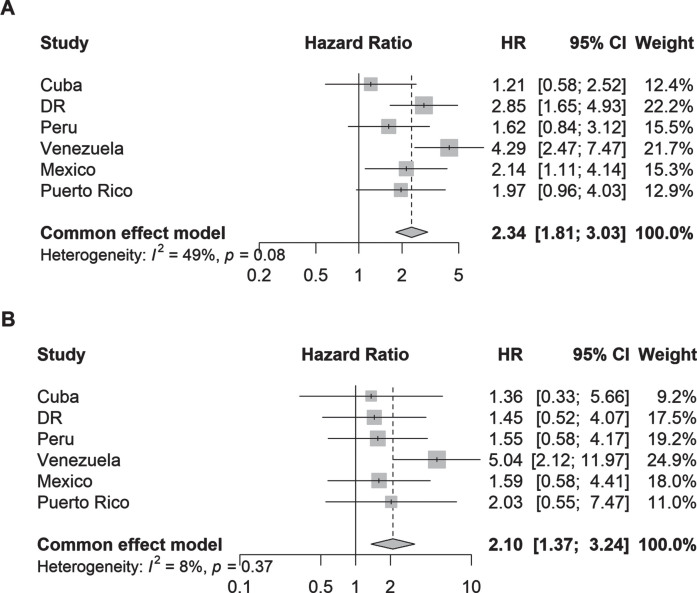
Hazard ratios (95% CI) of the association between (A) parkinsonism and (B) Parkinson’s disease and incident dependency by country. Cox proportional hazards regression models were stratified by education level and adjusted for age, sex, and the number of illnesses (none; 1-2 illnesses; 3 or more illnesses).

In the Cox proportional hazards models, both parkinsonism (adjusted hazard ratios [aHR] 2.34, 95% CI 1.81–3.03; I^2^ = 49%) and PD (aHR 2.10, 95% CI 1.37–3.24; I^2^ = 8%) were associated with more than 2-fold higher risk of incident dependency at follow-up (Fig. 3). Among individual countries, the strongest associations were detected in Venezuela for parkinsonism (aHR 4.29, 95% CI 2.47–7.47) and PD (aHR 5.04, 95% CI 2.12–11.97).

**Fig. 4:**
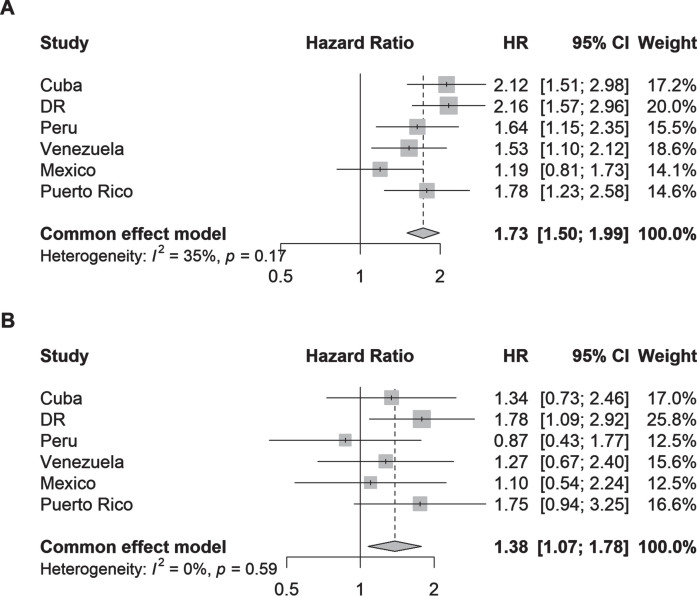
Hazard ratios (95% CI) of the association between (A) parkinsonism and (B) Parkinson’s disease and all-cause mortality by country. Cox proportional hazards regression models were stratified by the number of illnesses (none; 1-2 illnesses; 3 or more illnesses) and adjusted for age, sex, and education.

Parkinsonism (aHR 1.73, 95% CI 1.50–1.99; I^2^ = 35%) and PD (aHR 1.38, 95% CI 1.07–1.78; I^2^ = 0%) were also associated with higher risk of death within 4 years compared to no parkinsonism or PD (Fig. 4). Among individual countries, the strongest associations were found in Dominican Republic for parkinsonism (aHR 2.16, 95% CI 1.57–2.96) and PD (aHR 1.78, 95% CI 1.09–2.92).

As a sensitivity analysis, the main analysis was repeated for PD defined using the algorithm based on the current clinical criteria only (Supplementary Figure 2). The results were similar (somewhat attenuated) to when the PD definition was supplemented with self-reported diagnoses.

## DISCUSSION

### Summary of findings

To the best of our knowledge, the present study is the first to report the association between parkinsonism and PD with health service use, incident dependency, and all-cause mortality using a large prospective cohort of older adults from Latin America. The risk of incident dependency within 4 years was doubled in people with parkinsonism or PD compared to older people without parkinsonism or PD. The risk of dying was approximately 40%–70% higher. We also found that only parkinsonism was associated with higher odds of hospital admission and neither parkinsonism nor PD was associated with higher community health service use. There was low to moderate level of heterogeneity in estimates across countries, which may be partly explained by differences in healthcare systems. For instance, Puerto Rico had the highest level of community health service use (82%), which is likely due to their Medicare program that provides health insurance to individuals aged 65 and over [22]. Accordingly, we found that the only country with a significant association between PD and community health service use was Puerto Rico.

### Comparison with previous literature

Few papers were identified in the literature regarding the health service use of PD patients and generally restricted to Western countries. Previous studies in the US [5], UK [7], and Canada [6] found that PD patients had higher usage of health care in most categories (e.g., emergency admissions, rehabilitation service). While our study also found a positive association between PD and hospital admission, the association did not reach statistical significance likely due to the low number of PD cases.

Dependency is highly common in PD patients, in whom the likelihood of activities of daily living is 5 times greater than non-PD patients (RR 5.53, 95% CI 2.01–15.2) [23]. However, no studies, to our knowledge, studied the risk of incident dependency due to PD. A previous study in Serbia found no change in disability (based on the Self-Assessment Disability Scale [24]) after 2 years of follow-up and attributed this lack of change to the short follow-up [25]. Our findings provide novel piece of evidence that people living with PD who were previously not dependent have more than 2-fold greater risk of becoming dependent 4 years later.

The greater risk in mortality in people living with PD observed in the present study is also consistent with previous research conducted in Western countries. A systematic review identified 8 prospective studies of PD and all-cause mortality in the US or Europe [26]. Among 72,833 participants included in the meta-analysis, PD was associated with 2-fold higher risk of all-cause mortality (RR 2.22, 95% CI 1.78–2.77), [26] which is greater than our estimate (HR 1.40, 95% CI 1.09–1.80). The difference in the risk estimates between Western and Latin American settings suggests the potential for geographical variation in the risk of mortality by PD. These differences may be explained by differences in the cohort characteristics, such as age [26], sex [26, 27], disease duration [26], and differences in PD ascertainment [26]. For instance, a previous systematic review found that the association between PD and mortality was stronger among older people and males, which could be due to the higher rates of mortality in these populations [26]. Older people, due to their existing comorbidities and weakened physiological systems, may have greater vulnerability to PD and be at greater risk of hospitalization and death. The present study also ascertained PD using different methods. Only a third of the PD cases identified in our study had a previous PD diagnosis, which means that most of the PD cases were undiagnosed. Therefore, most cases were unlikely to be aware of their PD diagnosis and the proportion of PD cases receiving relevant medication is likely to be low, which may lead to greater risk of adverse outcomes in these cases. However, they may also be at an earlier, less severe stage of the disease, which may explain the weaker association with hospital admission and all-cause mortality observed in the present analysis than those found in Western studies. Also, true regional differences in the management of PD, such as the availability of PD medication [1], are likely to impact prognosis in PD.

### Strengths and limitations

The study used a large prospective, populated-based cohort, including older adults in six Latin American countries. Data were collected using systematic and standardized protocols and face-to-face structured interviews, which allowed comparison and pooling of results across study sites.

However, the study had some limitations. First, dependency was defined relatively subjectively; the interviewer determined participants’ need for care (some care versus much care) based on a semi-structured interview. There may be some variability in the assessment of dependency between interviewers, which may give rise to measurement error and underestimation of the association between PD and incident dependency. Thus, data on inter-rater reliability would have been useful but was not obtained. Second, attrition during follow-up is likely to result in the exclusion of participants who are more ill and dependent, which may affect the results of the present analyses. However, the differences in baseline characteristics of participants whose vital status were ascertained versus those whose were not ascertained was small with no significant differences in the rate of hospitalization and dependency. Some minor differences in age and community health service use were detected, but differences were small, so the exclusion of these participants were unlikely to have resulted in a large bias. Third, potentially important factors, such as awareness and use of PD medication, are likely to have influenced the association between PD and outcomes, but these measures were not available in the study. Lastly, the low number of PD cases resulted in the exclusion of certain countries from the meta-analysis and prevented authors from adjusting for other relevant factors, raising the potential risk of under adjustment of the associations.

### Implications for research and clinical practice

Our findings found significant burden associated with parkinsonism and PD, with cases having greater risk of hospital admission, dependency, and death. The enhanced risks suggest there is urgent need for further studies investigating the risk factors of adverse outcomes in PD in Latin America and interventions to alleviate this risk. While our study provides one of the first piece of evidence on the burden of PD in LMICs using a large, multinational cohort, the 10/66 study is over 10 years old. Thus, more recent studies should provide insight as to whether the increased awareness of disparities in the access to neurological care and medicines among LMICs [28], as well as efforts to address this, have had an impact on the burden of PD in areas like Latin America. This evidence should inform public health policies to reduce mortality and morbidities associated with PD.

Importantly, there is a paucity of evidence regarding the health service use among the PD population. In the present study, we found that people living with PD were no more likely to access community-based or hospital care or despite their diagnosis. This lack of association may be explained by several things. First, the cross-country comparison suggests that healthcare coverage is likely to be an important factor determining access to health service use among people living with PD like in the case of Puerto Rico. Second, only a small subset of the PD cases identified in our study had a previous PD diagnosis. Hence, the lack of association with community health service use by PD cases may be due to their lack of awareness of a diagnosis. Even among those with known PD diagnosis, patients have reported a preference for self-management of PD and specialists in PD [29]. Patients may feel that general practitioners (GPs) lack expert knowledge and skills due to the complexity of the disease [29]. However, recent research has suggested that early symptoms of PD may be detected in primary care settings as early as 10 years before diagnosis [30, 31] and GPs may provide a central role in referring patients to the correct multidisciplinary care [29, 32]. This highlights the potential importance of strengthening efforts to screen for and manage PD in primary care settings.

### Conclusion

Parkinsonism and PD is associated with hospital admission, incident dependency, and all-cause mortality in older adults living in Latin America after almost 4 years of average follow-up. Despite this, people living with parkinsonism or PD did not appear to seek any more community-based medical care compared to the general population. These findings highlight the need to enhance public health measures aimed at reducing morbidity and mortality related to PD. Further studies are also needed to understand the risk factors of adverse outcomes in parkinsonism and PD and assess the effectiveness of current approaches to manage PD in the community.

## Supplementary Material

Supplementary Material

## Data Availability

The data supporting the findings of this study are available on request from the corresponding author. The data are not publicly available due to privacy or ethical restrictions.
