# Supplementary material for: Burden of Parkinsonism and Parkinson’s Disease on Health Service Use and Outcomes in Latin America
Source: J Parkinsons Dis. 2023 Nov 3;13(7):1199–211. doi: 10.3233/JPD-230114 (PMC10657702; doi:10.3233/JPD-230114)
Supplement: Supplementary Material [file jpd-13-jpd230114-s001.pdf]

Supplementary Material

Burden of Parkinsonism and Parkinson's Disease on Health Service Use and Outcomes in Latin America

Supplementary Table 1. The STROBE Checklist

	Item No	Recommendation
**Title and abstract**	1	(*a*) Indicate the study's design with a commonly used term in the title or the abstract **page 3**
		(*b*) Provide in the abstract an informative and balanced summary of what was done and what was found **page 3**
**Introduction**		
Background/rationale	2	Explain the scientific background and rationale for the investigation being reported **page 5**
Objectives	3	State specific objectives, including any prespecified hypotheses **page 6**
**Methods**		
Study design	4	Present key elements of study design early in the paper **page 6-7**
Setting	5	Describe the setting, locations, and relevant dates, including periods of recruitment, exposure, follow-up, and data collection **page 7**
Participants	6	(*a*) Give the eligibility criteria, and the sources and methods of selection of participants. Describe methods of follow-up **page 6-7**
		(*b*) For matched studies, give matching criteria and number of exposed and unexposed **n/a**
Variables	7	Clearly define all outcomes, exposures, predictors, potential confounders, and effect modifiers. Give diagnostic criteria, if applicable **page 8-10**
Data sources/ measurement	8*	For each variable of interest, give sources of data and details of methods of assessment (measurement). Describe comparability of assessment methods if there is more than one group **page 8-10**
Bias	9	Describe any efforts to address potential sources of bias **page 10**
Study size	10	Explain how the study size was arrived at **page 6**
Quantitative variables	11	Explain how quantitative variables were handled in the analyses. If applicable, describe which groupings were chosen and why **page 9**
Statistical methods	12	(*a*) Describe all statistical methods, including those used to control for confounding **page 10**
		(*b*) Describe any methods used to examine subgroups and interactions **n/a**
		(*c*) Explain how missing data were addressed **page 11**
		(*d*) If applicable, explain how loss to follow-up was addressed **page 6**
		(*e*) Describe any sensitivity analyses **page 10**
**Results**		
Participants	13*	(a) Report numbers of individuals at each stage of study—e.g., numbers potentially eligible, examined for eligibility, confirmed eligible, included in the study, completing follow-up, and analyzed **page 40**
		(b) Give reasons for non-participation at each stage **page 40**
		(c) Consider use of a flow diagram **page 40**
Descriptive data	14*	(a) Give characteristics of study participants (e.g., demographic, clinical, social) and information on exposures and potential confounders **page 11-12**
		(b) Indicate number of participants with missing data for each variable of interest **page 34 for exposure**
		(c) Summarize follow-up time (e.g., average and total amount) **page 26**

Outcome data	15*	Report numbers of outcome events or summary measures over time **page 26, 35, 36, 38**
Main results	16	(*a*) Give unadjusted estimates and, if applicable, confounder-adjusted estimates and their precision (e.g., 95% confidence interval). Make clear which confounders were adjusted for and why they were included **page 27-30**
		(*b*) Report category boundaries when continuous variables were categorized **n/a**
		(*c*) If relevant, consider translating estimates of relative risk into absolute risk for a meaningful time period **n/a**
Other analyses	17	Report other analyses done—e.g., analyses of subgroups and interactions, and sensitivity analyses **page 41**
**Discussion**		
Key results	18	Summarize key results with reference to study objectives **page 14**
Limitations	19	Discuss limitations of the study, taking into account sources of potential bias or imprecision. Discuss both direction and magnitude of any potential bias **page 16-17**
Interpretation	20	Give a cautious overall interpretation of results considering objectives, limitations, multiplicity of analyses, results from similar studies, and other relevant evidence **page 15-18**
Generalizability	21	Discuss the generalizability (external validity) of the study results **page 17**
**Other information**		
Funding	22	Give the source of funding and the role of the funders for the present study and, if applicable, for the original study on which the present article is based **page 19-20**

Supplementary Table 2. The UK Parkinson's Disease Society Brain Bank diagnostic criteria

**Step 1. Diagnosis of Parkinsonism**	**Step 2. Exclusion criteria for Parkinson's disease**	**Step 3. Supportive criteria for Parkinson's disease (≥3 required for definitive diagnosis)**
Bradykinesia (slowness of initiation of voluntary movement with progressive reduction in speed and amplitude of repetitive actions) and at least one of the following:<br>• Muscular rigidity;<br>• 4–6 Hz rest tremor;<br>• Postural instability not caused by primary visual, vestibular, cerebellar or proprioceptive dysfunction.	• History of repeated strokes with stepwise progression of Parkinsonian features.<br>• History of repeated head injury.<br>• History of definite encephalitis.<br>• Oculogyric crises.<br>• Neuroleptic treatment at onset of symptoms.<br>• More than one affected relative.<br>• Sustained remission.<br>• Strictly unilateral features after three years.<br>• Supranuclear gaze palsy.<br>• Cerebellar signs.<br>• Early severe autonomic involvement.<br>• Early severe dementia with disturbances of memory, language and praxis.<br>• Babinski sign.<br>• Presence of a cerebral tumor or communicating hydrocephalus on CT scan.<br>• Negative response to large doses of levodopa (if malabsorption excluded).<br>• MPTP exposure.	• Unilateral onset.<br>• Rest tremor present.<br>• Progressive disorder.<br>• Persistent asymmetry affecting the side of onset most.<br>• Excellent response (70–100%) to levodopa.<br>• Severe levodopa-induced chorea.<br>• Levodopa response for 5 years or more.<br>• Clinical course of 10 years or more.

MPTP, 1-methyl-4-phenyl-1,2,3,6-tetrahydropyridine. Table produced using information from Clarke et al. [1].

[1] Clarke CE, Patel S, Ives N, Rick CE, Woolley R, Wheatley K, Walker MF, Zhu S, Kandiyali R, Yao G, Sackley CM, Group on behalf of the PRC (2016) UK Parkinson's Disease Society Brain Bank Diagnostic Criteria. In *Clinical effectiveness and cost-effectiveness of physiotherapy and occupational therapy versus no therapy in mild to moderate Parkinson's disease: a large pragmatic randomised controlled trial (PD REHAB) NIHR Journals Library*.

Supplementary Table 3. Cross-tabulation of the Parkinson's disease diagnosis by diagnosis source

	UKPDSBB non-case	UKPDSBB case	UKPDSBB missing
**Self-reported PD non-case**	1822	61	235
**Self-reported PD case**	72	13	15
**Self-reported PD missing**	9656	156	835

Total number of PD cases equal the sum of green numbers (61+72+13+15+156=317) and the total number of PD non-cases equal the sum of black numbers (1822+235+9656=11713). 835 participants missing data on UKPDSBB and self-reported PD were excluded from the logistic and Cox regression analyses.

PD, Parkinson's disease; UKPDSBB, UK Parkinson's Disease Society Brain Bank diagnostic criteria.

Supplementary Table 4. Dependency cohort characteristics at baseline, overall and by individual country.

N (%) or Mean (SD)	Overall	Cuba	Dominican Republic	Peru	Venezuela	Mexico	Puerto Rico
Total	7,296	1,059	1,140	1,265	1,217	1,413	1,202
**Age**	73.51 (6.50)	73.32 (6.22)	73.77 (6.66)	74.03 (6.89)	71.46 (6.21)	73.56 (6.17)	74.86 (6.33)
**Male sex**	2,496 (34.2)	355 (33.5)	352 (30.9)	485 (38.3)	428 (35.2)	503 (35.6)	373 (31.1)
**Marital status**							
Never married	540 (7.4)	81 (7.7)	80 (7.0)	140 (11.1)	100 (8.3)	70 (5.0)	69 (5.8)
Married/cohabiting	3,584 (49.4)	500 (47.3)	363 (32.0)	733 (58.4)	614 (51.0)	741 (52.5)	633 (52.8)
Widowed	2,245 (30.9)	281 (26.6)	447 (39.3)	330 (26.3)	324 (26.9)	508 (36.0)	355 (29.6)
Divorced/ separated	893 (12.3)	194 (18.4)	246 (21.7)	53 (4.2)	166 (13.8)	93 (6.6)	141 (11.8)
**Education level**							
None	801 (11.0)	28 (2.7)	219 (19.3)	78 (6.2)	80 (6.6)	366 (25.9)	30 (2.5)
Some, did not complete primary	1,996 (27.5)	207 (19.6)	571 (50.3)	157 (12.5)	250 (20.7)	612 (43.4)	199 (16.6)
Completed primary	2,135 (29.4)	327 (31.0)	219 (19.3)	476 (38.0)	633 (52.5)	260 (18.4)	220 (18.3)
Completed secondary	1,446 (19.9)	276 (26.1)	83 (7.3)	338 (27.0)	183 (15.2)	97 (6.9)	469 (39.0)
Tertiary (college)	880 (12.1)	218 (20.6)	43 (3.8)	203 (16.2)	60 (5.0)	76 (5.4)	280 (23.3)
**Number of assets**							
1st quartile – least assets	1,106 (15.2)	154 (14.6)	317 (27.9)	96 (7.6)	30 (2.5)	244 (17.3)	265 (22.0)
2nd quartile	2,711 (37.2)	323 (30.7)	269 (23.7)	713 (56.4)	791 (65.0)	615 (43.5)	0 (0.0)
3rd quartile	1,959 (26.9)	318 (30.2)	441 (38.8)	143 (11.3)	0 (0.0)	120 (8.5)	937 (78.0)
4th quartile – most assets	1,510 (20.7)	257 (24.4)	110 (9.7)	313 (24.7)	396 (32.5)	434 (30.7)	0 (0.0)
**Number of illnesses**							
No illnesses	2,940 (40.4)	442 (41.8)	337 (29.6)	588 (46.6)	495 (40.9)	618 (43.7)	460 (38.4)
One to two illnesses	3,143 (43.2)	524 (49.6)	563 (49.5)	523 (41.4)	431 (35.6)	570 (40.3)	532 (44.4)
Three or more illnesses	1,197 (16.4)	91 (8.6)	238 (20.9)	152 (12.0)	285 (23.5)	225 (15.9)	206 (17.2)
**Parkinsonism**	404 (5.9)	38 (3.6)	80 (7.2)	85 (6.8)	39 (4.2)	96 (6.8)	66 (6.2)
**Parkinson's disease**	135 (2.0)	21 (2.0)	23 (2.1)	26 (2.1)	24 (2.5)	25 (1.8)	16 (1.5)
**Dependency at follow-up**	536 (7.3)	79 (7.5)	115 (10.1)	75 (5.9)	82 (6.7)	72 (5.1)	113 (9.4)

The dependency cohort included participants with no need for care at baseline, who were reinterviewed during the incidence phase, and did not have missing dependency data.

Supplementary Table 5. Mortality cohort characteristics at baseline, overall and by individual country.

N (%) or Mean (SD)	Overall	Cuba	Dominican Republic	Peru	Venezuela	Mexico	Puerto Rico
Total	10,315	1,749	1,706	1,752	1,697	1,844	1,567
**Age**	74.60 (7.18)	74.85 (6.91)	75.35 (7.57)	74.72 (7.38)	72.34 (6.83)	74.24 (6.59)	76.26 (7.24)
**Male sex**	3,642 (35.3)	584 (33.4)	574 (33.7)	677 (38.6)	625 (36.8)	669 (36.3)	513 (32.8)
**Marital status**							
Never married	803 (7.8)	148 (8.5)	120 (7.1)	189 (10.8)	156 (9.4)	95 (5.2)	95 (6.1)
Married/cohabiting	4,845 (47.3)	742 (42.6)	512 (30.2)	1011 (58.0)	827 (49.9)	948 (51.4)	805 (51.5)
Widowed	3,330 (32.5)	544 (31.2)	686 (40.5)	463 (26.6)	455 (27.5)	691 (37.5)	491 (31.4)
Divorced/ separated	1,262 (12.3)	308 (17.7)	376 (22.2)	79 (4.5)	219 (13.2)	109 (5.9)	171 (10.9)
**Education level**							
None	1,174 (11.5)	45 (2.6)	328 (19.4)	106 (6.1)	135 (8.1)	508 (27.6)	52 (3.3)
Some, did not complete primary	2,958 (28.9)	391 (22.5)	883 (52.3)	214 (12.3)	364 (21.9)	808 (43.9)	298 (19.0)
Completed primary	2,989 (29.2)	563 (32.3)	307 (18.2)	650 (37.4)	847 (51.0)	315 (17.1)	307 (19.6)
Completed secondary	1,915 (18.7)	403 (23.1)	110 (6.5)	479 (27.6)	234 (14.1)	117 (6.4)	572 (36.5)
Tertiary (college)	1,193 (11.7)	339 (19.5)	60 (3.6)	287 (16.5)	82 (4.9)	94 (5.1)	331 (21.1)
**Number of assets**							
1st quartile – least assets	1,742 (16.9)	298 (17.1)	540 (31.7)	124 (7.1)	43 (2.5)	337 (18.3)	400 (25.5)
2nd quartile	3,760 (36.5)	453 (26.0)	383 (22.5)	1031 (58.8)	1107 (65.2)	786 (42.6)	0 (0.0)
3rd quartile	2,728 (26.5)	606 (34.8)	628 (36.9)	171 (9.8)	0 (0.0)	156 (8.5)	1167 (74.5)
4th quartile – most assets	2,072 (20.1)	384 (22.1)	150 (8.8)	426 (24.3)	547 (32.2)	565 (30.6)	0 (0.0)
**Number of illnesses**							
No illnesses	3,967 (38.6)	686 (39.4)	491 (28.8)	796 (45.5)	662 (39.7)	776 (42.1)	556 (35.6)
One to two illnesses	4,433 (43.2)	881 (50.5)	798 (46.8)	716 (40.9)	604 (36.2)	753 (40.8)	681 (43.6)
Three or more illnesses	1,870 (18.2)	176 (10.1)	415 (24.4)	238 (13.6)	401 (24.1)	315 (17.1)	325 (20.8)
**Parkinsonism**	770 (8.1)	115 (6.6)	180 (11.1)	128 (7.3)	75 (6.1)	165 (9.0)	107 (8.0)
**Parkinson's disease**	241 (2.5)	46 (2.6)	46 (2.8)	37 (2.1)	38 (2.9)	44 (2.4)	30 (2.2)
**Mortality**	1,730 (16.8)	404 (23.1)	467 (27.4)	152 (8.7)	200 (11.8)	209 (11.3)	298 (19.0)

Supplementary Table 6. Baseline status of participants by whether their vital status was ascertained.

	Vital status NOT ascertained	Vital status ascertained	p
**N**	2,550	10,315	
**Age (mean (SD))**	75.32 (7.42)	74.60 (7.18)	<0.001
**Gender = male (%)**	926 (36.3)	3,642 (35.3)	0.352
**Marital status (%)**			<0.001
never married	241 (9.5)	803 (7.8)	
married/ cohabiting	1,000 (39.4)	4,845 (47.3)	
widowed	915 (36.1)	3,330 (32.5)	
divorced/ separated	382 (15.1)	1,262 (12.3)	
**No. of illnesses (%)**			<0.001
no illnesses	1,099 (43.2)	3,967 (38.6)	
one to two illnesses	1,034 (40.6)	4,433 (43.2)	
three or more illnesses	412 (16.2)	1,870 (18.2)	
**One or more community medical services accessed (%)**	1,399 (54.9)	5,906 (57.3)	0.031
**Hospitalization (%)**	83 (3.3)	296 (2.9)	0.345
**Dependency (%)**			0.334
needs care much of the time	168 (6.7)	595 (6.0)	
needs care some of the time	113 (4.5)	476 (4.8)	
does not need care; they are able to do everything for them	2,224 (88.8)	8,926 (89.3)	

Supplementary Table 7. Prevalence of outcomes by baseline status of parkinsonism and Parkinson's disease.

	Parkinsonism			Parkinson's disease		
N (%)	No	Yes	p	No	Yes	p
**Baseline cohort (N=12,865)**	10,851	934		11,713	317	
One or more community health service use*	6,072 (56.0)	545 (58.4)	0.168	6,576 (56.1)	198 (62.5)	0.029
Hospital admission*	266 (2.5)	40 (4.3)	0.001	323 (2.8)	8 (2.5)	0.931
**Dependency cohort (N=7,296)**	6,439	404		6,761	135	
Incident dependency**	403 (6.3)	83 (20.5)	<0.001	481 (7.1)	25 (18.5)	<0.001
**Mortality cohort (N=10,315)**	8,738	770		9,461	241	
Deaths	1,247 (14.3)	260 (33.8)	<0.001	1,544 (16.3)	66 (27.4)	<0.001

*Health service utilization 3 month prior to interview at baseline

**Incident dependency defined as needing care much of the time at interview during the incident phase in those without dependency at baseline

UKPDSBB, United Kingdom Parkinson's Disease Society Brain Bank

Supplementary Table 8. Odds ratios (95% CI) of the association parkinsonism and Parkinson's disease with health service use for the 10/66 participants at baseline

**Health service use in the last 3 months**	**Parkinsonism**	**Parkinson's disease**
	OR (95% CI)	OR (95% CI)
Any community health service use	1.02 (0.89-1.18)	1.18 (0.93-1.49)
Primary care	0.90 (0.76-1.07)	1.31 (1.01-1.69)
Government hospital doctor	1.07 (0.90-1.27)	0.86 (0.64-1.14)
Other government health worker	1.05 (0.73-1.48)	0.71 (0.35-1.27)
Private doctor	1.14 (0.97-1.34)	1.06 (0.81-1.37)
Dentist	0.87 (0.64-1.15)	0.88 (0.54-1.36)
Traditional healer	0.88 (0.34-1.89)	0.44 (0.02-1.99)
Hospital admission	1.52 (1.06-2.15)	0.77 (0.35-1.47)

Logistic regression models were adjusted for age, sex, education, and the number of illnesses (none; 1-2 illnesses; 3 or more illnesses).

Supplementary Figure 1. Flow diagram of study participants

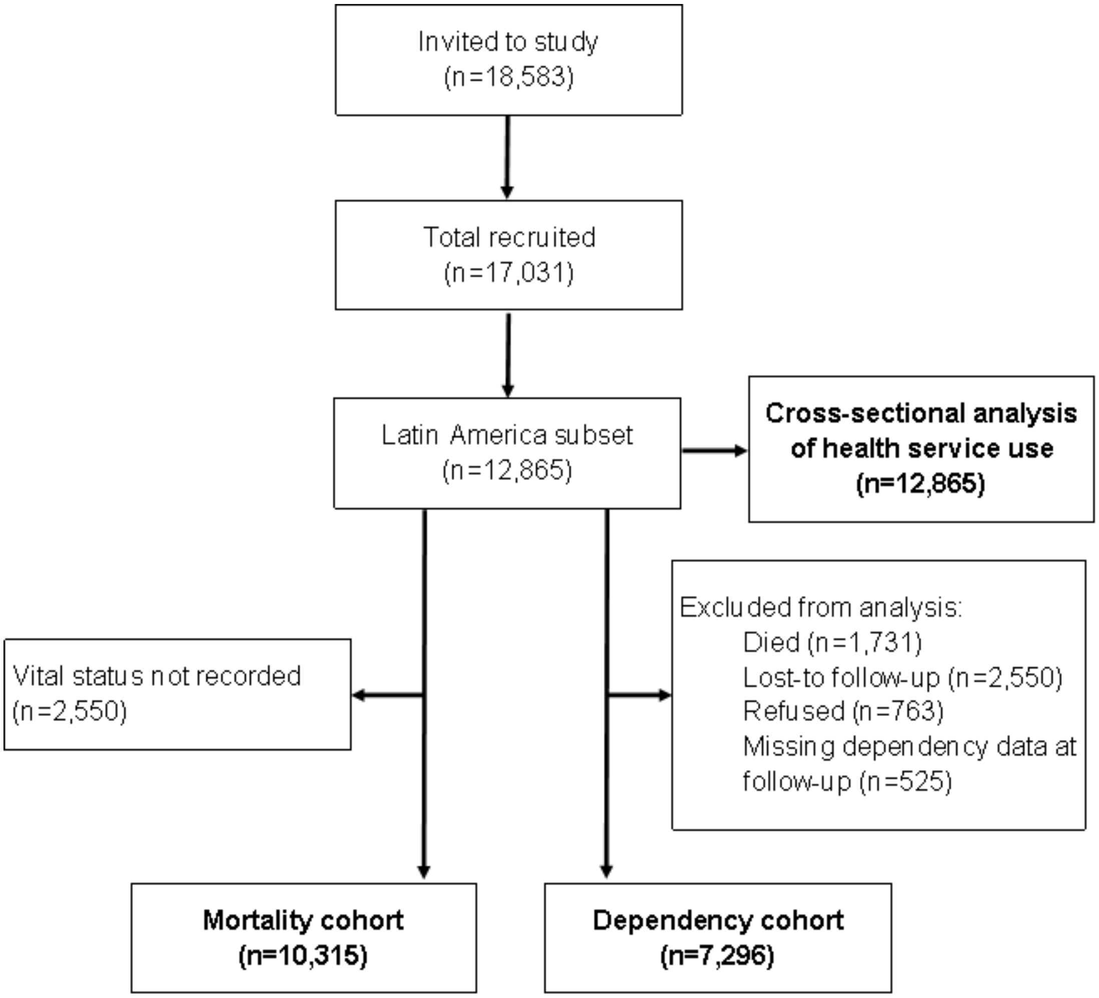

Supplementary Figure 2. Odds and hazard ratios (95% CI) of the association between Parkinson's disease (excluding self-reported diagnoses) and (A) community health service use, (B) hospital admission, (C) incident dependency, and (D) all-cause mortality by country.

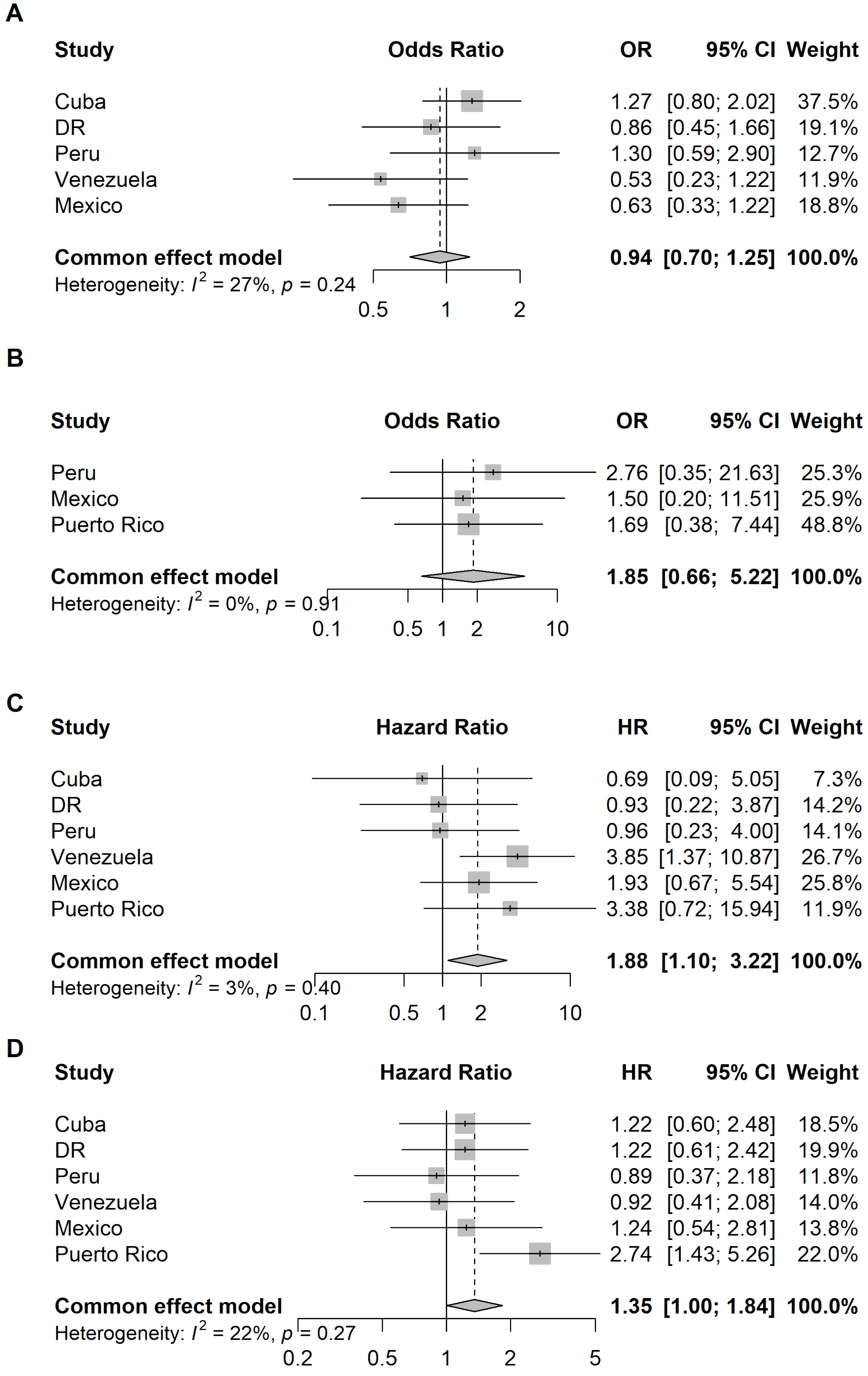

Plot A and B: Logistic regression models were adjusted for age, sex, education, and number of illnesses; Plot A excludes data from Puerto Rico and Plot B excludes data from Cuba, Dominican Republic, and Venezuela due to zero events, thus data from these countries did not contribute to the fixed-effects model; Plot C: Cox regression models were stratified by education level and adjusted for age, sex, and the number of illnesses; Plot D: Cox regression models were stratified by the number of illnesses and adjusted for age, sex, and education.
